# Behavioral and functional connectivity basis for peer-influenced bystander participation in bullying

**DOI:** 10.1093/scan/nsy109

**Published:** 2018-11-27

**Authors:** Kyosuke Takami, Masahiko Haruno

**Affiliations:** 1 Center for Information and Neural Networks, NICT, Suita, Osaka, Japan; 2 Graduate School of Frontier Biosciences, Osaka University, Suita, Osaka, Japan

**Keywords:** bullying, conformity, social anxiety, amygdala, TPJ, resting-state fMRI

## Abstract

Recent studies have shown that the reactions of bystanders who witness bullying significantly affect whether the bullying persists. However, the underlying behavioral and neural mechanisms that determine a peer-influenced bystander’s participation in bullying remain largely unknown. Here, we designed a new ‘catch-ball’ task where four players choose to throw a sequence of normal or strong (aggressive) balls in turn and examined whether the players (*n* = 43) participated in other players’ bullying. We analyzed behaviors with a computational model that quantifies the tendencies of a participant’s (i) baseline propensity for bullying, (ii) reactive revenge, (iii) conformity to bullying, and (iv) capitulation to threat and estimated these effects on the choice of balls. We found only conformity had a positive effect on the throwing of strong balls. Furthermore, we identified a correlation between a participant’s conformity and social anxiety. Our mediation analysis of resting-state functional magnetic resonance imaging revealed that there were significant relationships of each participant’s functional connectivity between the amygdala and right temporoparietal junction (TPJ) and social anxiety to the participant’s conformity to bullying. We also found that amygdala–TPJ connectivity partially mediated the relationship between social anxiety and conformity. These results highlighted the anxiety-based conformity and amygdala network on peer-influenced bystander participation in bullying.

## Introduction

Bullying is an aggressive behavior with the intention of doing repeated harm in an interpersonal relationship and is an increasingly serious problem for many communities (Olweus, 1994, 1995). Witnesses are present in most bullying incidents, and their reactions influence the bullying (Lynn Hawkins *et al.*, 2001). That is, when bystanders participate in the bullying, the bully is more likely to continue (Kärnä *et al.*, 2008). Therefore, understanding the underlying behavioral and neural mechanisms of the peer-influenced bystander’s participation in bullying would help the development of preventative measures. However, although several previous studies have experimentally measured individual-level aggression (Bandura *et al.*, 1961; Taylor, 1967), few studies have attempted to measure group aggression (Meier *et al.*, 2007), particularly bullying.

Previous questionnaire-based studies have proposed three main reasons why people bully. The most emphasized is that bullies have low empathy (Endresen and Olweus, 2001; Jolliffe and Farrington, 2006; Gini *et al.*, 2007; Caravita *et al.*, 2009; Jolliffe and Farrington, 2011) compared with their peers. Second, it has been suggested that the baseline propensity for bullying plays a role. Bullying itself may act as a reward to the bully, as it potentially contributes to a higher status (Pellegrini and Long, 2002; Salmivalli and Peets, 2009). Finally, bullies were reported to have higher anxiety and fear (Swearer *et al.*, 2001; Kelleher *et al.*, 2008). In addition to these main issues, reactive revenge to frustration may be a reason for bullying (Dollard *et al.*, 1939; Berkowitz, 1962). However, because these studies were based on questionnaires in classrooms and did not involve behavioral tasks or neural investigations, the relative importance of the above factors and their neural implementations remain unknown.

**Fig. 1:**
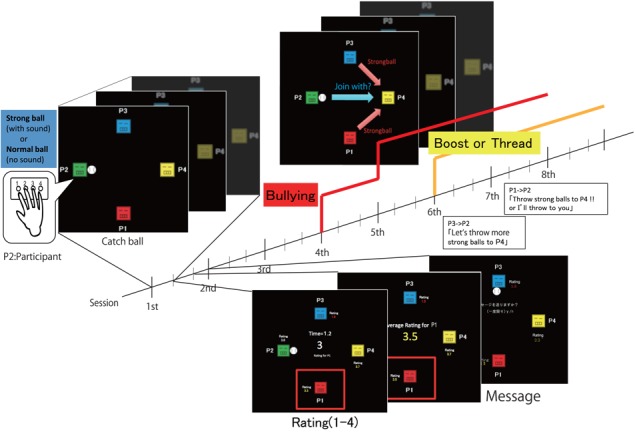
Task design of the catch-ball task. Participants are assigned a player number (P1–P4). Each player can throw a ball of two different strengths (normal or strong) to any of the other three players. Strong balls are associated with an unpleasant sound that could mildly injure the recipient player. In reality, all participants are unknowingly assigned P2, and the actions of P1, P3 and P4 are controlled by a computer program. One session consists of eight ball throws and at the end of each session, each player rates the other players (1–4 scale). Participants can send messages to other players in all sessions. In session 3, P4 begins to throw strong balls with an equal probability to the other players. In session 4, P1 starts to throw strong balls to P4 repeatedly. In session 5, P3 starts to throw strong balls to P4 repeatedly, and P1 continues to do so. In sessions 6 and 7, participants receive boost or threat messages from P1 or P3 such as ‘Let’s throw more strong balls to P4’ or ‘Throw strong balls to P4 or I’ll throw them to you’.

In the present study, to examine the behavioral and neural mechanisms for peer-influenced bullying behavior, we designed a new ‘catch-ball’ task where four participants sitting at different desks equipped with a desktop computer chose to throw a normal or strong ball in turn to any of the other three players (Figure 1). Strong balls were associated with an unpleasant sound and were mildly harmful to the recipient player. Although we instructed the four participants that they would play the game as different players, all of them were unknowingly assigned the role of Player 2 (P2: participant) and played against three computer-programmed players (P1, P3 and P4: computer; see also Supplementary Figure S1).

The catch-ball setting was first introduced in the well-established ‘Cyberball’ (Williams *et al.*, 2000), which was used to study social rejection in the laboratory (Twenge *et al.*, 2001, 2003, 2007; Eisenberger *et al.*, 2003; Baumeister *et al.*, 2005; Eisenberger, 2012). We used a similar catch-ball setting to dissociate the following factors associated with bullying: (i) the baseline propensity for bullying, (ii) reactive revenge (Dollard *et al.*, 1939; Berkowitz, 1962), (iii) conformity to bullying, and (iv) capitulation to threat. The baseline propensity can be measured as a participant’s tendency to throw strong balls over all sessions without any specific context. In session 3, one player (P4) begins throwing strong balls to the other players in revenge for the behaviors of P1 and P3 in session 2 (see Materials and methods). An instant increase of strong balls to P4 by the participant (P2) can be regarded as reactive revenge. Then, in sessions 4 and 5, P1 and/or P3 throw strong balls to P4 repeatedly. Therefore, P2 throwing strong balls to P4 in response to the behavior of P1 and P3 indicates conformity to bullying. Finally, in sessions 6 and 7, P2 receives threat messages. P2 throwing strong balls in response to these messages is capitulation to a threat. We defined a utility function based on the game session and normal and strong ball throws to different players (the meaning of strong and normal balls depends on the game session and the target player), where the above four factors are incorporated as linear components.

We also contrasted these factors with the participants’ personality traits and functional connectivity in the brain as measured by resting-state functional magnetic resonance imaging (fMRI), which is now widely used to investigate generic neural networks for personality traits such as empathy and anxiety (Cox *et al.*, 2012; Smith *et al.*, 2013; Sampaio *et al.*, 2014; Modi *et al.*, 2015; Oathes *et al.*, 2015), as well as intrinsic connectivity networks such as the well-known default mode network (Buckner *et al.*, 2008; Raichle, 2015) and for biomarkers of psychiatric disorders (Greicius, 2008; Castellanos *et al.*, 2013; Drysdale *et al.*, 2016). Accordingly, we compared bullying behavior measures with resting-state fMRI connectivity as a biomarker of bullying behavior.

Among the potential behavioral mechanisms behind peer-influenced bystander participation in bullying, we are particularly interested in anxiety, since some of recent questionnaire-based investigations of bullying suggested that not only victims but also bullies tend to be anxious (Swearer *et al.*, 2001; Kelleher *et al.*, 2008). As possible underlying neural substrates, the amygdala has been implicated in anxiety (Davis, 1992; De Bellis *et al.*, 2000; Davidson, 2002; Rauch *et al.*, 2003; Roy *et al.*, 2009; Cisler and Koster, 2010). Therefore, we hypothesized that connectivity including the amygdala may be involved in peer-influenced bystander participation in bullying in relation to anxiety.

## Materials and methods

### Participants

Informed consent was obtained from all participants, and the experiment protocol was approved by the ethics committees of the National Institute of Information Technology. Forty-three male undergraduate and graduate students aged 20–26 (21.6 ± 1.5 years) participated in both the behavioral and resting-state fMRI experiments. For each participant, two experiments were conducted on different days, separated by at least 72 h. As such, no transfer was expected between the two experiments. All participants were males, because males were reported to be more aggressive than females (Maccoby and Jacklin, 1974; Mitchell, 1981) in physical settings such as the ‘catch-ball’ task. Before the experiments, we measured each participant’s Interpersonal Reactivity Index (IRI; Davis, 1983) for empathy and social anxiety, and Big Five Inventory (BFI; John and Srivastava, 1999) for other measures of their personality traits.

### Behavioral tasks

Four participants were invited into an experiment room together and sat at different desks equipped with a desktop computer and a display. The catch-ball task consisted of eight sessions (Figure 1). Each player could throw a ball of two different strengths (normal or strong) to any of the other three players. We instructed participants that strong balls can harm other players. In fact, strong balls move faster than normal balls on the screen and are associated with an unpleasant sound that is like the one used in fighting games and mildly harmful to the recipient player. Moreover, when a participant receives a strong ball, the participant cannot throw a strong ball in the next throw.

Although we instructed the four participants that they would play the game as different players, all of them were unknowingly assigned the role of Player 2 (P2: participant) and played against three computer-programmed players (P1, P3 and P4: computer; see also Supplementary Figure S1). Since the participants did not know each other’s assigned number, player anonymity was maintained. In each session, after the participants threw eight balls in total, the four participants were asked to rate the other players’ behavior within the session on a scale of 1–4 (1 = bad, 4 = good). More specifically, the catch-ball game proceeded as follows (see also Figure 1):

- Players can throw a ball of two different strengths: strong or normal.
- The player who receives a strong ball cannot throw a strong ball in the next throw.
- Players throw a normal ball to another player by pressing the key which corresponds to the player number only once or a strong ball by pressing the key twice.
- After each catch-ball session (eight throws in total), players are asked to rate the other players’ behavior by pressing a key (1 = bad, 4 = good) within 3 s. The average rating is displayed at the center of the screen in the order of P1 to P4 until the next session begins.
- Players can send messages of shorter than 20 Japanese words to the other players after the rating in each session. Messages can be read only by the recipient player.

### Behavior of computer-programmed players

Players P1, P3 and P4 were controlled by a computer program and followed the behavioral strategy described below in each of the eight sessions (see also Supplementary Figure S1):

Session 1: Throw normal balls to other players with equal probability.
Session 2: P1 and P3 never throw a ball to P4.
Session 3: P4 throws strong balls to other players with equal probability. In contrast, P1 and P3 throw only normal balls.
Session 4: P1 throws only strong balls to P4.
Session 5: P1 and P3 throw only strong balls to P4.
Session 6: Prior to this session, P2 receives the message from P3, ‘Let’s throw more strong balls to P4’.
Session 7: If P2 never threw a strong ball to P4 in session 6, he receives the message ‘Throw strong balls to P4 or I’ll throw them to you’ from P1, and P1 and P3 throw only strong balls to P2 and P4. Otherwise, P2 receives the message ‘Let’s throw more strong balls to P4’ from P1, and P1 and P3 continue to throw only strong balls to P4.
Session 8: P1 and P3 continue to throw strong balls the same way as in session 7.

### Model-based analysis.

To analyze each participant’s peer-influenced participation in bullying quantitatively, we defined the utility function, *U*(*X_t_*), based on normal and strong ball throws to different players in each trial as equation (1). *U*(*X_t_*) contains not only effects (i.e. **βi**) but also dummy variables, (**f**_**i**_**(X**_**t**_**)**), which allow us to represent different meanings of strong and normal balls depending on the session and target player. For example, conformity to bullying, the key variable of interest in our analysis, is linked with the dummy coding *f_3_*(*X_t_*). After session 3, a normal ball to P4 is considered direct help to P4, and *f_3_*(*X_t_*) is set to −1. In contrast, a normal ball to P1 or P3 may represent indirect aggression to P4 (rather than neutral), and *f_3_*(*X_t_*) is set to 0.5, whereas a strong ball to P4 is direct aggression, and therefore *f_3_*(*X_t_*) is set to 1.

(1)' \begin{align*} U(X_t)=&\ \beta 0+\beta 1\ f_{1}(X_{t})+\beta 2\ \textrm{react}(t)\,f_2(X_t)+\beta 3\ \textrm{conf}(t)\,f_3(X_t)\nonumber\\ &+\beta 4\ \textrm{message}(t)\,f_4(X_t)+\beta 5\ {\rm total\_strong\_balls}(t)\,f_5(X_t)\end{align*}

β0: intercept, β1: baseline propensity for bullying, β2: reactive revenge, β3: conformity to bullying, β4: capitulation to threat and β5: accumulation effect of previous strong balls by

\begin{align*}f_{1}(X_{t}) =&\ \begin{cases}\ \quad1:&\mathrm{Xt}=\mathrm{S}1,\mathrm{S}3,\mathrm{S}4\\ {}-1:&\mathrm{Xt}=\mathrm{N}1,\mathrm{N}3,N4\end{cases}\\[-2pt]  f_{2}(X_{t})=&\ \begin{cases}\quad1:& Xt=S4\\\  -1:& Xt=\left(N1,N3,N4\right)\ \textrm{or}\ \left(S1,S3\right)\ \end{cases}\end{align*}

\begin{align*}f_{3}(X_{t}) =&\ \begin{cases}\quad1:&\mathrm{Xt}=\kern0.5em \mathrm{S}4\\ \,0.5:&\mathrm{Xt}=\mathrm{N}1,\mathrm{N}3\\ \ -1:& \mathrm{Xt}=\left(\mathrm{N}4\right)\ \mathrm{or}\ \left(\mathrm{S}1,\mathrm{S}3\right)\end{cases}\\ f_{4}(X_{t}) =&\ \begin{cases}\quad1:&\mathrm{Xt}=\mathrm{S}4\\ \,-1:& \mathrm{Xt}=\left(\mathrm{N}1,\mathrm{N}3,\mathrm{N}4\right)\ \mathrm{or}\ \left(\mathrm{S}1,\mathrm{S}3\right)\end{cases}\\ f_{5}(X_{t}) =&\  \begin{cases}\ 1:& \mathrm{Xt}=\mathrm{S}1,\mathrm{S}3,\mathrm{S}4\\ \ 0:&\mathrm{Xt}=\mathrm{N}1,\mathrm{N}3,\end{cases}\end{align*}

S: strong ball, N: normal ball (i.e. S4 means strong ball to P4).

\begin{align*}\textrm{react}(t)=&\ \begin{cases}1:&\mathrm{only}\ \mathrm{session}\ 3\\ 0:&\mathrm{other}\ \mathrm{session}\mathrm{s}\end{cases}\\ \textrm{conf}(t)=&\  \begin{cases} 0:&\mathrm{session}\mathrm{s}\ 1,2,3\\ 1:&\mathrm{session}\ 4\\  2:&\mathrm{session}\ 5,6,7,8\end{cases}\\ \textrm{message}(t)= &\ \begin{cases} 0:&\mathrm{sessions}\ 1,2,3,4,5\\ 1:&\mathrm{sessions}\ 6,7,8\end{cases}\end{align*}

total_strong_balls(t): total number of strong balls from all players from the beginning of the task to trial t.

*U*(*X_t_*) (X_t_ is N1, N3, N4, S1, S3 or S4; for instance, N1 stands for a normal ball to P1) contains six linear coefficients (parameters, β0, β1, β2, β3, β4 and β5), which represent the contributions of different factors on bystander participation in bullying. Because we need to distinguish ball throws to P4 from those to P1 or P3 due to their different meanings, **X**_**t**_ takes four values (N1 = N3, N4, S1 = S3 and S4).

When β1 is large, the participant tends to throw strong balls over all sessions independent of the session number or any specific context [see the definition of f_1_(Xt) above]. β2 quantifies whether the participant seeks revenge to strong balls in session 3 or not (Dollard *et al.*, 1939; Berkowitz, 1962). The react(t) function in equation (1)' takes value 1 only in session 3, when participants receive strong balls from P4. β3 quantifies how closely the participant conforms to the bullying behavior of P1 and P4. The conf(t) function in equation (1)' represents the strength of pressure to conform. From sessions 1 to 3, conf(t) takes a value of 0; in session 4, conf(t) is 1, since only P1 throws strong balls to P4; and from session 5 onward, conf(t) becomes 2, since both P1 and P3 throw strong balls to P4. When participants throw a normal ball to P1 or P3, f_3_(Xt) takes a value of 0.5, because normal balls to P1 or P3 assist the bullying to P4 indirectly. β4 quantifies how much a participant contributed to the bullying in response to the threating message. We defined message(t) as the strength of the threat. In sessions 1–5, message(t) takes a value of 0; in sessions 6–8, message(t) becomes 1, since participants receive messages in sessions 6 and 7. Finally, β5 represents the effect of previous strong balls, in which total_strong_balls(t) is the total number of strong balls by all players from the beginning of the task to trial t. Because total_strong_balls(t) monotonically increases from the first to last sessions (Supplementary Figure S3A), β5 also reflects the session effect.

To investigate the session (trial) effect more directly, we also performed an analysis that replaced total_strong_balls(t) with trial_number(t), as shown in equation (1′). trial_number(t) represents the present trial number and equals the total number of balls thrown (including both strong and normal) up to that trial.

(1) \begin{align*} U(X_t)=&\ \beta 0+\beta 1\ f_{1}(X_{t})+\beta 2\ \textrm{react}(t)\,f_2(X_t)+\beta 3\ \textrm{conf}(t)\,f_3(X_t)\nonumber\\ &+\beta 4\ \textrm{message}(t)\,f_4(X_t)+\beta 5\ {\rm trial\_number}(t)\,f_5(X_t)\qquad\ \ \,(1^\prime)\end{align*}

The results for the analysis with (1′) are shown in Supplementary Figure S3B.

We estimated the six parameters (β0–β5; denoted as vector $\boldsymbol{\theta}$) for each participant from their ball throws, *X_t_*, by the maximum likelihood estimation method of *U*(*X_t_*). Therefore, the minimization procedure of the negative log-likelihood of the participant’s behavior (D; i.e. a set of $Xt$) is identical to the multinomial logit model (Train, 2009), as shown in equations (2), (3) and (4). In (2), β is a free parameter known as the inverse temperature parameter or slope and is also determined by the maximum likelihood estimation. β1 in equation (1)' represents the bias toward a strong or normal ball. This non-linear minimization of the negative log-likelihood was conducted by a standard technique (Daw, 2011) using the MATLAB function ‘fmincon’.

(2) \begin{equation*} \mathrm{P}(Xt)=\frac{\exp \left(\beta \cdotp U(Xt)\right)}{\sum_{Xc=N1,N3,N4}^{S1,S3,S4}\exp \left(\beta \cdotp U(Xc)\right)} \end{equation*}

(3) \begin{equation*} \min - \log\ P (D |\boldsymbol{\theta})\end{equation*}

(4) \begin{equation*} P(D | \boldsymbol{\theta})= {\prod}_tP(Xt) \end{equation*}

It may be argued that the non-linear optimization function ‘fmincon’ may result in a suboptimal solution. Therefore, we compared three optimization algorithms: the inter-point, sqp and active-set methods starting with different initial values. We obtained the same average negative likelihoods and parameter values from all settings (Supplementary Table S1). These results suggested that the estimated values are optimal.

### Questionnaires

We conducted two questionnaires: the IRI and the BFI. The IRI consists of 28 items answered on a 5-point Likert scale. The measure has four subscales: **Perspective Taking (P score)**, which measures the tendency to spontaneously adopt the other’s point of view; **Fantasy (F score)**, which measures the tendency of the subject to shift themselves imaginatively to the feelings and actions of fictitious characters in books, movies and plays; **Empathic Concern (E score)**, which assesses other-oriented feelings of sympathy and concern for unfortunate others; and **Personal Distress (D score)**, which assesses the feelings of anxiety and unease in tense interpersonal settings. The BFI consists of 70 items that measure the Big Five Factors (dimensions) of personality: extraversion, agreeableness, neuroticism, openness and intelligence.

#### Functional magnetic resonance imaging (fMRI) data processing

Structural and resting-state fMRI scans were performed using a 3 T (Siemens Magnetom Trio A Tim System) MRI scanner at the Center for Information and Neural Network (CiNet, Osaka, Japan) with a 32 channel head coil. Functional images were acquired with a gradient echo-planar imaging (EPI) sequence of T2^*^-weighted images (TR/TE/flip angle: 2500/30/80; FOV: 192 mm; voxel size: 3.0×3.0×3.0 mm) during an 8 min rest condition, when participants were instructed to keep their eyes open and fixate. In addition, a high-resolution (1.0×1.0×1.0 mm) structural scan was acquired from each participant with a T1-weighted MPRAGE sequence.

Although many studies have used an atlas-based definition such as the Brodmann-based AAL (Uylings *et al.*, 2005; Achard *et al.*, 2006), this definition may not represent any of the constituent time courses if different functional areas are included within a single node. The regions of interest (ROIs) we used were data-driven functional ROIs produced from the resting-state fMRI data of 79 healthy participants and parcellated by group-wise graph theory-based analysis (https://www.nitrc.org/frs/?group_id=51; Functional Brain Atlas from Shen *et al.*, 2013). We focused on brain structures related to decision-making and emotion, and excluded sensory, motor and visual cortices (i.e. the cerebellum, visual, auditory, motor and somatosensory areas) from the ROIs. As Shen’s ROIs did not separate the amygdala, we adopted more finely divided definitions (i.e. amygdalostriatal (AStr), centro-medial (CM), latero-basal (LB) and superficial (SF) that were taken from SPM Anatomy toolbox (http://www.fz-juelich.de/inm/inm-1/DE/Forschung/_docs/SPMAnatomyToolbox/SPMAnatomyToolbox_node.html, see also Supplementary Table S2).

Functional connectivity was analyzed with ROI-to-ROI correlation mapping using the CONN toolbox 18.a (web.conn-toolbox.org) based on SPM (Wellcome Department of Imaging Neuroscience, London, UK) since the removal of artefacts is a crucial first step of the resting-state fMRI analysis. Spatial preprocessing of the CONN toolbox included realignment, normalization and smoothing (8 mm FWHM Gaussian filter) using SPM12 default parameter settings. Anatomical volumes were segmented into gray matter, white matter and cerebrospinal fluid (CSF) areas, and the resulting masks were eroded to minimize partial volume effects. The temporal time series characterizing the estimated subject motion (three-rotation and three-translation parameters, plus another six parameters representing their fist-order temporal derivatives and scrubbing parameters containing the offending scans), as well as the blood-oxygen-level dependent (BOLD) fMRI time series within the subject-specific white matter mask [three principal component analysis (PCA) parameters] and the CSF mask (three PCA parameters) were used as temporal covariates and removed from the BOLD functional data using linear regression. The resulting residual BOLD time series were then band-pass filtered (0.008 Hz < f < 0.10 Hz).

Pearson correlation coefficients between the time courses of each possible pair of nodes were calculated and normalized to Z scores using the Fisher transformation, resulting in a 146 × 146 symmetrical connectivity matrix for each participant (ROI-to-ROI analysis in CONN). After this basic processing, Pearson correlation coefficients between a participant’s connectivity matrix and the participant’s behavior parameters (i.e. β1, β2, β3, β4 and β5) were computed. To visualize brain network links, we used BrainNet Viewer (Xia *et al.*, 2013) (http://www.nitrc.org/projects/bnv/).

### Mediation analysis

To quantify and test whether resting-state fMRI connectivity might affect the effect of the Personal Distress scores on conformity (β3), we performed a standard mediation analysis using a mediation tool box (https://github.com/canlab/MediationToolbox) (Wager *et al.*, 2008). This analysis quantifies the degree to which a relationship between two variables, *X* and *Y*, can be explained by another variable, *M*. We defined *X* as Personal Distress scores, *Y* as conformity (β3) and *M* as resting-state fMRI connectivity (Figure 4C). Paths **a** and **b** measure the association between Personal Distress scores and the mediator (resting-state connectivity) and also the association between mediator and conformity (β3) while controlling for Personal Distress, respectively. More specifically, path **b** tests whether resting-state fMRI connectivity predicts variations in conformity that are conditionally independent of Personal Distress.

On the other hand, Paths **c** and **c’** respectively measure the total relationship between Personal Distress and conformity including direct and indirect effects and also the direct effect of the relationship between Personal Distress and conformity controlling for resting-state connectivity. Finally, product a*b tests the significance of the mediators. We conducted bootstrap tests (10 000 iterations) for statistical significance of the mediators.

### Post-experiment questionnaire about behavioral task

After the behavioral and resting-state fMRI experiments were done, we sent the following post-experiment questionnaires about the behavioral task to all 43 participants by postal mail. These questionnaires were administered via postal mail because the experimenter had to handle four participants in behavioral experiment, and fMRI experiments were tightly scheduled, which did not permit direct follow-up with the participants. We analyzed the psychological effect of strong balls and the percentage of participants who noticed that other players (i.e. P1, P3 and P4) were controlled by a computer program. The questionnaire was written as follows (translated from Japanese):

*Please answer the following questions regarding the experiment entitled ‘The experiment of others’ behavior and evaluation’*, which *was done at NICT. In this experiment, four participants came in a room and played catch ball with each other on the PC.*

1. *In the catch-ball task, players could throw two different strengths of balls, normal and strong. When you received a strong ball with a sound, what was your reaction in comparison with a normal ball? Choose one option.*

(a) *Negative*
(b) *Positive*
(c) *Neutral*

2. *Did you have any sense of unnatural behavior from the other players during the task?*
(a) *Yes*
(b) *No*

3. *If you chose ‘Yes’, please describe what was unnatural as detailed as possible.*

### Experimental design and statistical analysis

The statistical design of our experiment can be found in the following Results section. We used Matlab functions as a statistical software to perform the analyses.

## Results

### Behavioral experiment

**Fig. 2:**
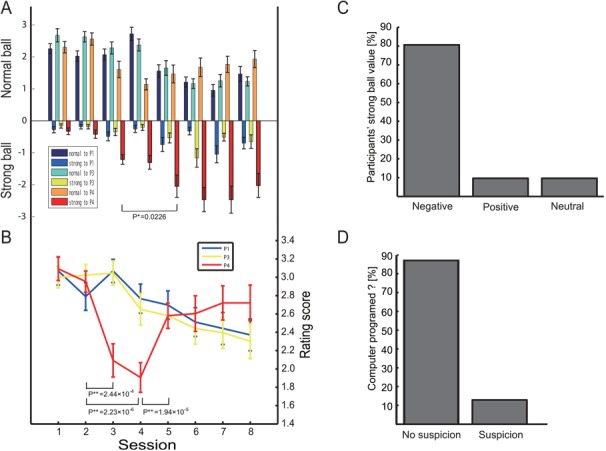
Player behaviors and ratings, and post-experiment questionnaires. (**A**) Bar graphs show the mean ball types (normal or strong) per session. Normal balls are shown in the positive region and strong balls in the negative region. From session 3 to session 5, the number of strong balls thrown by P2 to P4 increased significantly (*F* (2,42) = 3.32, *P* < 0.05), showing the effect of conformity. (**B**) Participants’ ratings of the other players. In session 3, the rating of P4 significantly decreased compared to the rating in session 2 (one sample t-test, *P* = 2.44 × 10^−4^). Error bars represent standard errors. (**C**) More than 80% of participants felt negatively toward strong balls. (**D**) Almost 90% of participants did not suspect that other players were computer-programmed.

The ball direction and strength averaged over all participants are illustrated in Figure 2A. In sessions 1 and 2, participants threw few strong balls. However, in session 3, in which P4 started to throw strong balls with an equal probability to the other players, the number of strong balls thrown to P4 by P2 increased. There was a significant effect of sessions 3, 4 and 5 on the number of strong balls thrown by P2 to P4 [*F* (2,42) = 3.32, *P* < 0.05]. A post-hoc analysis showed that the number of strong balls thrown by P2 to P4 in session 5 was significantly larger than that in session 3 [one sample t-test, *t*(42) = 2.37, *P* = 0.0226]. Thereafter, the average number of strong balls thrown at P4 by P2 in each session stayed higher than 1. These data demonstrate that participants conformed to the bullying behavior of P1 and P3.

Figure 2B plots the mean participants’ ratings of the other players. The rating of P4 significantly decreased to ~2.1 in session 3 [one sample t-test, *t*(42) = 4.01, *P* = 2.44 × 10^−4^, in comparison with session 2], which was the time when P4 significantly increased the number of strong balls thrown to the other three players. In addition, our post-experiment questionnaire confirmed that >80% of participants had a negative value for strong balls (Figure 2C, see also *Post-experiment questionnaire about behavioral task* in Materials and methods). These results indicated that strong balls had negative effects and aggressive meaning to the participants. The rating remained low in session 4 [one sample t-test, *t*(42) = 5.48, *P* = 2.23 × 10^−6^, in comparison with session 2]. However, in session 5, the rating dramatically recovered to ~2.6 [one sample t-test, *t*(42) = −4.81,*P* = 1.94 × 10^−5^, in comparison with session 4] and remained stable for the rest of the experiments. Interestingly, we can see that the bullying of P4 lasted even after the rating of P4 recovered to that in session 2.

Finally, one may argue that the participants might have noticed the programmed nature of the computer programmed players. Our post-experiment questionnaire revealed that almost 90% of participants did not show suspicion (Figure 2D, see also *Post-experiment questionnaire about behavioral task* in Materials and methods). Furthermore, although four participants reported they suspected that the other players were computer programmed in the questionnaire, the results of the behavioral and resting-state fMRI analyses did not change even if the data of these participants were excluded (Supplementary Figure S2).

### Computational model-based analysis

**Fig. 3:**
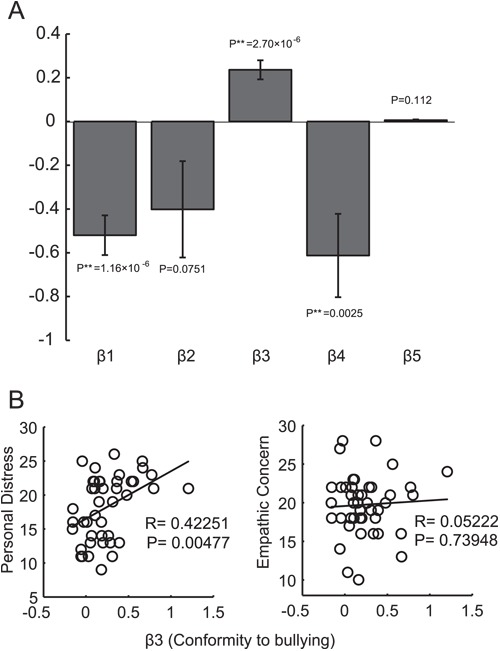
Estimates of β1, β2, β3, β4 and β5. (**A**) Bars show the mean estimates of β1, β2, β3, β4 and β5. (**B**) Correlation of IRI scores with β3 (Conformity). β3 had a significantly positive correlation with the Personal Distress score (left) but not with the Empathic Concern score (right).

To investigate the participants’ bullying behavior more quantitatively, we adopted a computational model that includes five parameters in the utility function: β1 (baseline propensity for bullying), β2 (reactive revenge), β3 (conformity to bullying), β4 (capitulation to threat) and β5 (effect of previous strong balls). In addition to these, we also included β (slope in equation (2)) (see also Materials and methods). We estimated these parameters for each participant by the maximum likelihood estimation method based on P2 (participant) ball throws to P1, P3 and P4 (Figure 3).

**Fig. 4:**
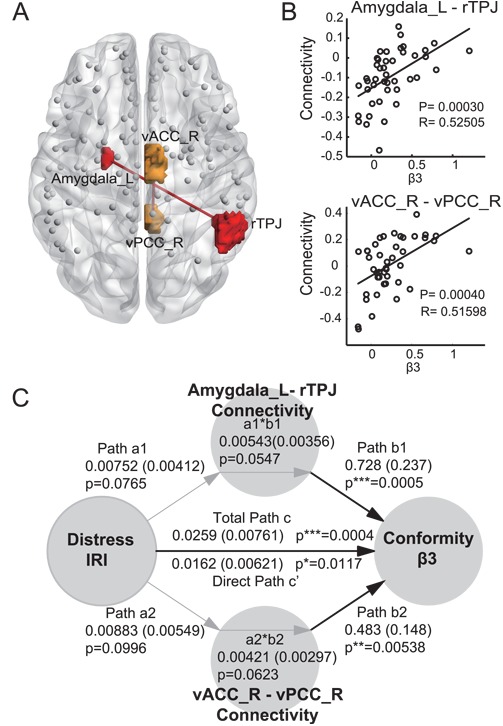
Correlation of brain network links with behavioral parameters and personality traits. β3 (Conformity) had a positive correlation with brain network links. (**A**) Red edges show brain network links that correlated with β3 (*P* < 0.0005). (**B**) Each participant’s strength of connectivity and β3 (similar to Figure 3B). (**C**) Mediation analysis of the functional connectivity on the link from Personal Distress to Conformity (β3). Path coefficients are shown next to arrows with standard errors in parentheses. Paths **a1 a2** and **b1 b2** represent the relationship of Personal Distress to Connectivity, and from Connectivity to Conformity while controlling for Personal Distress. Path **c’** represents the direct relationship from Distress to Conformity controlling for Connectivity, and **c** represents the total relationship of Personal Distress to Conformity (not adjusted for any other factors). P^***^ < 0.001, P^**^ < 0.01, P^*^ < 0.05, two-tailed. Black and gray arrows show significant and marginally significant (0.05 < *P* < 0.1) relationships, respectively.

We found β3 was significantly positive, while β1 and β4 were significantly negative [Figure 3A; one sample t-test, β1;*t*(42) =−5.71, *P* < 0.0001, β2;*t*(42) = −1.83, *P* = 0.0751, β3;*t*(42) = 5.42, *P* < 0.0001, β4;*t*(42) = −3.21, *P* < 0.01, β5;*t*(42) = 1.63, *P* = 0.112]. These data demonstrate that conformity is a driving force in peer-influenced bystander participation in bullying (β3), that a participant’s default action selection is a normal ball (β1) and that a participant does not participate in bullying in response to a threat (β4). In this analysis, the estimated values of β (slope; mean = 2.7223, s.d. = 0.2573) were comparable among participants. We also conducted the analysis in which the total number of strong balls was replaced with the session number. The results were highly consistent with Figure 3A (Supplementary Figure S3B) and showed that neither the number of strong balls nor trials had a positive effect on a participant throwing a strong ball. Therefore, it is conformity that increased strong ball throws rather than the habituation to strong balls.

We then investigated whether personality traits are related to conformity to bullying (i.e. β3). We were particularly interested in empathy and anxiety, which were measured by using the IRI because previous studies emphasized the contribution of the former over the latter to bullying (Endresen and Olweus, 2001; Jolliffe and Farrington, 2006, 2011; Gini *et al.*, 2007; Caravita *et al.*, 2009). We found a significant positive correlation between β3 (conformity to bullying) and Personal Distress (D score; Figure 3B, left, *R* = 0.42251, *P* = 0.00477, slope = 6.9973), which represents social anxiety or unease. Consistent with this finding, β3 (conformity to bullying) also had a positive correlation with neuroticism in the BFI (*R* = 0.36878, *P* = 0.01496, slope = 12.7405).

These data show that social anxiety or unease is correlated with conformity to bullying. In addition, β3 (conformity to bullying) also had a marginally negative correlation with Perspective Taking (*R* = −0.37970, *P* = 0.01203, slope = −4.7511), which relates to the mind-reading of others. We did not find any significant correlations between β1 (baseline propensity for bullying), β2 (reactive revenge), β3 (conformity to bullying), β4 (capitulation to threat) or β5 (effect of previous strong balls) and Empathic Concern (Figure 3B, right), which represents empathy. These findings differ from previous studies that used questionnaires and reported the importance of empathy in bullying (Endresen and Olweus, 2001; Jolliffe and Farrington, 2006, 2011; Gini *et al.*, 2007; Caravita *et al.*, 2009).

To seek the reason for this discrepancy, we conducted a resting-state fMRI analysis of the same participants. For each participant, behavioral and resting-state fMRI experiments were conducted on different days, and they were separated by at least 72 h. It is therefore unlikely the experiments affected each other.

**Table 1:** Brain connectivity strength that correlated with β3 (Conformity) (*P* < 0.0005)

Node	MNI			Node	MNI			*R*	*P*	slope
Amygdala_L (CM)	−21	−6	−14	rTPJ	51	−46	40	0.52505	0.00030	0.2500
vACC_R	7	−6	43	vPCC_R	6	−42	19	0.51598	0.00040	0.3629

### Resting-state fMRI functional connectivity results

We calculated functional ROI-to-ROI connectivity with the CONN tool box and 146 ROIs (Supplementary Table S2) based on the state-of-the-art functional brain atlas (Shen *et al.*, 2013), which is constructed from healthy-population resting-state fMRI data (see Materials and methods). Pearson correlation coefficients between the time courses of each possible pair of nodes were calculated and used to construct 146×146 symmetrical connectivity matrices, where each element defines the connection strength of an edge between two nodes. A connectivity matrix was constructed for each participant, and the Pearson correlation coefficients between the elements of the matrix and the corresponding participant’s behavioral parameter β3 (conform to bullying) was computed (we set the threshold of the mean Pearson correlation coefficient > 0.4, and the statistical threshold *P* < 0.0005, which corresponded to Bonferroni corrected *P* < 0.05).

We found two positively correlated links with β3 (conformity to bullying): connections between the left amygdala (Medial Centroid) and the right temporoparietal junction (TPJ; *P* = 0.00030 and *R* = 0.53), and the right ventral anterior cingulate cortex (vACC_R) and the ventral posterior cingulate cortex (vPCC_R) (*P* = 0.00040 and *R* = 0.52; Figure 4A and B; Table 1).

To investigate the relationship between the functional connectivity and anxiety-based conformity to bullying, we performed a mediation analysis, which determines whether the relationship between Personal Distress and conformity is mediated by connectivity between the amygdala and the rTPJ, and between vACC and vPCC (Figure 4C). First, the total relationship between Personal Distress and conformity was highly significant (the coefficient for path **c** = 0.0259, *z* = 0.00761, *P* = 0.0004), indicating that increased conformity is associated with more anxiety. Both of the two functional connectivities were associated with conformity highly significantly after controlling for Personal Distress (the coefficient for path **b1** = 0.728, *z* = 0.237, *P* = 0.0005, and path **b2** = 0.483, *z* = 0.148, *P* = 0.00538), indicating that the two functional connectivities are deeply involved in conformity. In addition, anxiety was also linked marginally with connectivity between the amygdala_L and rTPJ (coefficient for path **a1** = 0.00752, *z* = 0.00412, *P* = 0.0765), and connectivity between the vACC and vPCC (coefficient for path **a2** = 0.00883, *z* = 0.00549, *P* = 0.0996). The mediation effect of the two functional connectivities were marginally significant (a1^*^b1, coefficient = 0.00543, *z* = 0.00356, *P* = 0.0547, a2^*^b2 = 0.00421, *z* = 0.00297, *P* = 0.0623).

## Discussion

In the present study, we introduced the catch-ball task to examine the behavioral and neural mechanisms behind peer-influenced bystander participation in bullying. We analyzed the effects of a participant’s (i) baseline propensity for bullying, (ii) reactive revenge, (iii) conformity to bullying, (iv) capitulation to threat and (v) effect of previous strong balls, and found that only conformity had a significantly positive effect on a bystander throwing strong balls. Furthermore, there was a significant correlation between a participant’s conformity to bullying and a participant’s Personal Distress (i.e. social anxiety and unease), but not Empathy Concern. Consistent with these results, resting-state fMRI analysis showed that a participant’s conformity to bullying is correlated with the strength of the functional connectivity between the amygdala and TPJ, and the right vACC and the vPCC. Finally, mediation analysis showed that two functional connectivities partially modulate the links from social anxiety to conformity to bullying. These results revealed an important role of anxiety-based conformity and its underlying brain network in peer-influenced bystander participation in bullying.

Several behavioral studies have successfully examined individual-level aggression (Bandura *et al.*, 1961; Taylor, 1967), but far fewer have examined group aggression (Meier *et al.*, 2007), particularly bullying. To measure peer-influenced bystander participation in bullying, we devised a computer-programmed catch-ball setting, which was originally adopted in the Cyberball task to investigate the psychological effects (Twenge *et al.*, 2001, 2003, 2007; Baumeister *et al.*, 2005) and underlying neural substrates (Eisenberger *et al.*, 2003, 2012) of victims of social isolation. Similarly, our catch-ball task, which focuses on bullying, also showed psychological effects (e.g. Figure 2A–C) and a sense of reality (Figure. 2D). It may be argued that the strong balls we adopted here are insufficiently aversive to be comparable with real-life bullying. However, it is reported that aggression often begins at a low level, such as teasing or cursing, and escalates to more injurious behavior (Taylor *et al.*, 1979; Goldstein *et al.*, 1981). Therefore, we think that this task is appropriate for investigating the early stage of bullying.

Many questionnaire-based studies have emphasized that affectively empathic people tend to be less involved in bullying (Warden and Mackinnon, 2003; Gini *et al.*, 2007; Caravita *et al.*, 2009; Pöyhönen *et al.*, 2010). However, no correlation was found between the affective empathy scale and bullying-controlling parameters (i.e. β1, β2, β3, β4 and β5) in the present study. Instead, correlation was found between the conformity to bullying (i.e. β3) and social anxiety. An association between victimization such as bullying and social anxiety was also reported in previous questionnaire-based studies (Swearer *et al.*, 2001; Kelleher *et al.*, 2008). Differences between questionnaire responses and the present task behavior may be explained by the distinction between automatic emotional judgments involved only in task behavior and reflective cognitive judgements that play a major role in questionnaires. Consistent with the view from an automatic emotional response, our resting-state fMRI analysis revealed that functional connectivity between emotional brain structures, such as the amygdala and anterior cingulate cortex, was correlated with the conformity to bullying (i.e. β3).

It has been shown repeatedly that the amygdala plays an important role in fear and anxiety processing (Davis, 1992; Davidson, 2002; Cisler and Koster, 2010; Fox and Kalin, 2014). In particular, the central amygdala is proposed to be the integrative hub for anxiety (Gilpin *et al.*, 2015). These studies are consistent with our results since the amygdala_L ROI we used corresponds with the central amygdala.

Co-activation of the left amygdala and rTPJ was reported in several previous studies, although human rTPJ is thought to be anatomically connected with the right amygdala and not the left (Pitcher *et al.*, 2017). A resting-state fMRI study reported that functional connectivity between the left amygdala and bilateral limbic and somatomotor cortices positively correlated with state anxiety scores (He *et al.*, 2016). Additionally, the left amygdala and rTPJ were activated when individuals competed against a familiar friend in an episodic encoding task (Sugimoto *et al.*, 2016). Other research on children and adolescents showed that perceiving others being harmed was associated with increased hemodynamic activity in the left amygdala and rTPJ (Yoder *et al.*, 2016).

Similar to the amygdala, many studies established that both the anterior cingulate cortex (Osuch *et al.*, 2000; Etkin *et al.*, 2011; Barthas *et al.*, 2015) and posterior cingulate cortex (Milad *et al.*, 2013) are involved in the processing of anxiety. These previous studies are consistent with our observation that functional connectivity amygdala_L – rTPJ and vACC – vPCC partially mediated social anxiety and conformity to bullying.

Social conformity plays a key role in human social behavior and has been intensively studied in previous fMRI studies (Asch, 1956; Klucharev *et al.*, 2009; Campbell-Meiklejohn *et al.*, 2010; Edelson *et al.*, 2011; Toelch and Dolan, 2015). Specifically, conflict with group opinion was associated with activity in the rostral medial prefrontal cortex (Klucharev *et al.*, 2009), ventral striatum (Klucharev *et al.*, 2009; Campbell-Meiklejohn *et al.*, 2010) and amygdala and hippocampus (Edelson *et al.*, 2011). However, conformity is likely a broader concept containing not only value-based decision-making (Asch, 1956; Toelch and Dolan, 2015) and memory (Edelson *et al.*, 2011) but also a variety of emotions (Asch, 1956; Toelch and Dolan, 2015) including social anxiety. Therefore, the functional connectivity between the amygdala and rTPJ we found may provide novel insights into the neural mechanisms for anxiety-based behavioral changes under the influence of others.

Although anxiety, in particular on the bully side, has not attracted much attention in previous studies on bullying, some studies based on questionnaires have reported that not only victims but also bullies tend to be anxious and depressed (Swearer *et al.*, 2001; Kelleher *et al.*, 2008). Our behavioral and resting-state fMRI results successfully highlighted the quantitative relationship between bullying and anxiety from the behavioral and neuroscientific points of view. Recently, it was reported that the application of a Finnish anti-bullying intervention program called Kiva (Salmivalli *et al.*, 2011) not only reduced bullying but also decreased the level of anxiety in students (Williford *et al.*, 2012). Future work on anti-bullying programs should clarify the relationship between anti-bullying interventions focusing on anxiety and anxiety-related brain activity. Such investigation would open up a new neuroscientific methodology to evaluate the effectiveness of such programs.

In summary, the present study demonstrated an important role of anxiety-based conformity and its underlying neural networks that link the amygdala and rTPJ and the vACC and vPCC in peer-influenced bystander participation in bullying. These findings suggest the possibility that a person’s reliance on a group may contribute to preventing the spread of bullying and that behavioral and neuroscientific studies on bullying can contribute to the development of anti-bullying measures in the future.

## Supplementary Material

scan-18-227-File006_nsy109

scan-18-227-File007_nsy109

scan-18-227-File008_nsy109

Supplementary_Tables_nsy109
