# Supplementary figures and images for: Behavioral and functional connectivity basis for peer-influenced bystander participation in bullying
Source: Soc Cogn Affect Neurosci. 2018 Nov 27;14(1):23–33. doi: 10.1093/scan/nsy109 (PMC6348439; doi:10.1093/scan/nsy109)

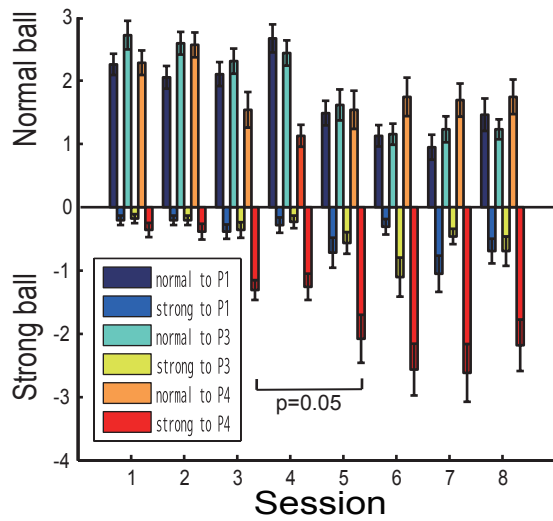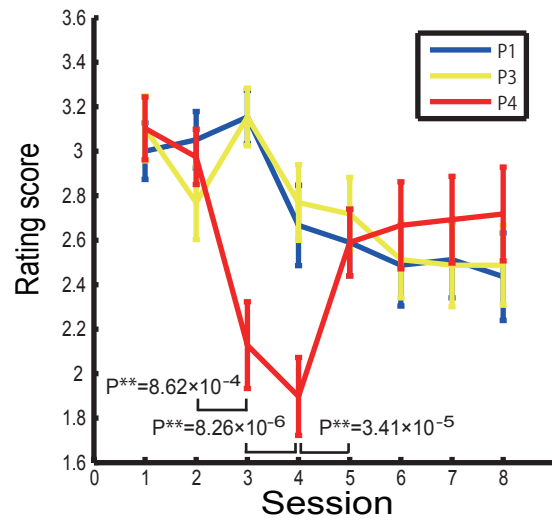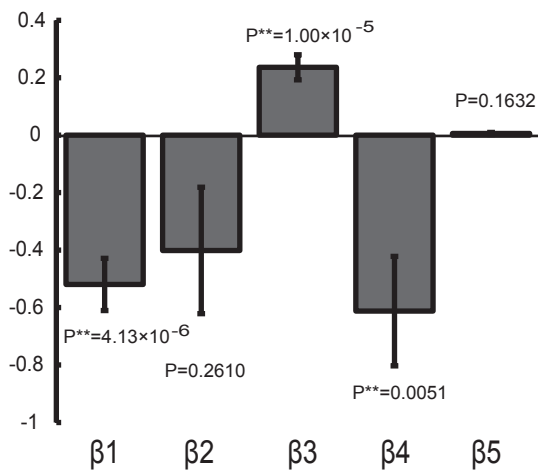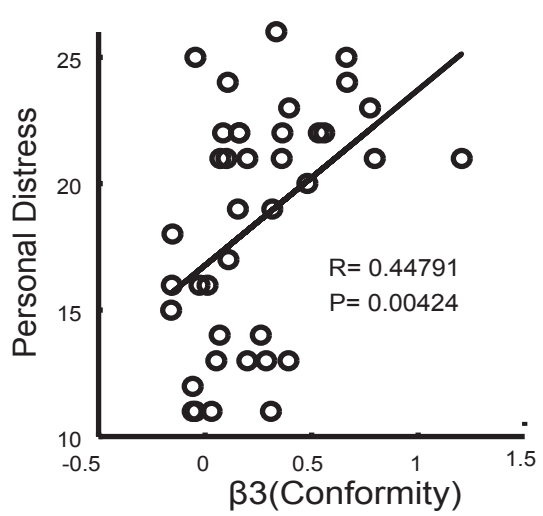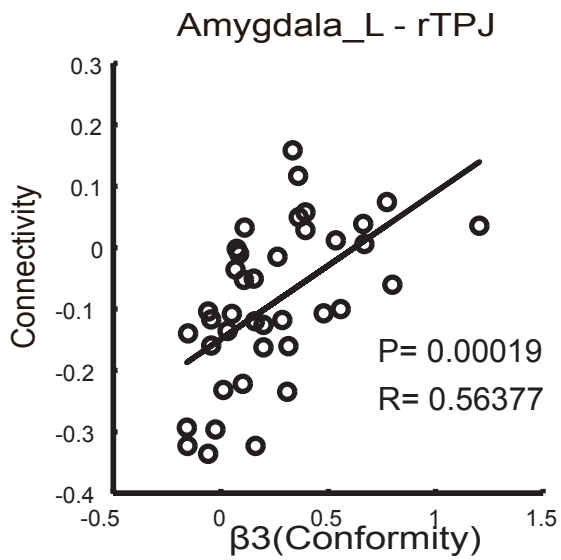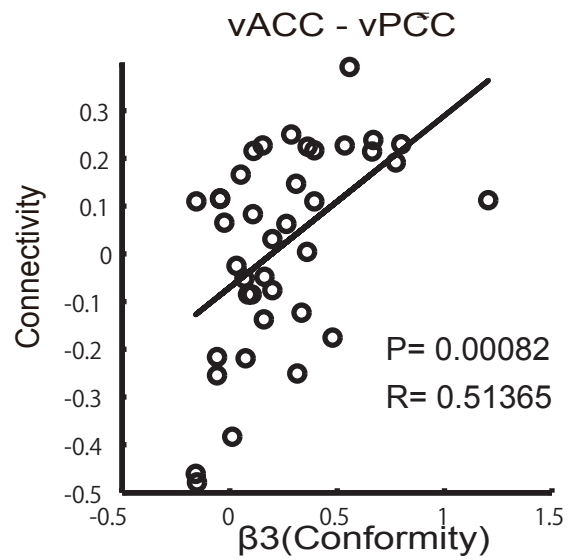

Supplement: scan-18-227-File007_nsy109 [file scan-18-227-file007_nsy109.pdf]

A

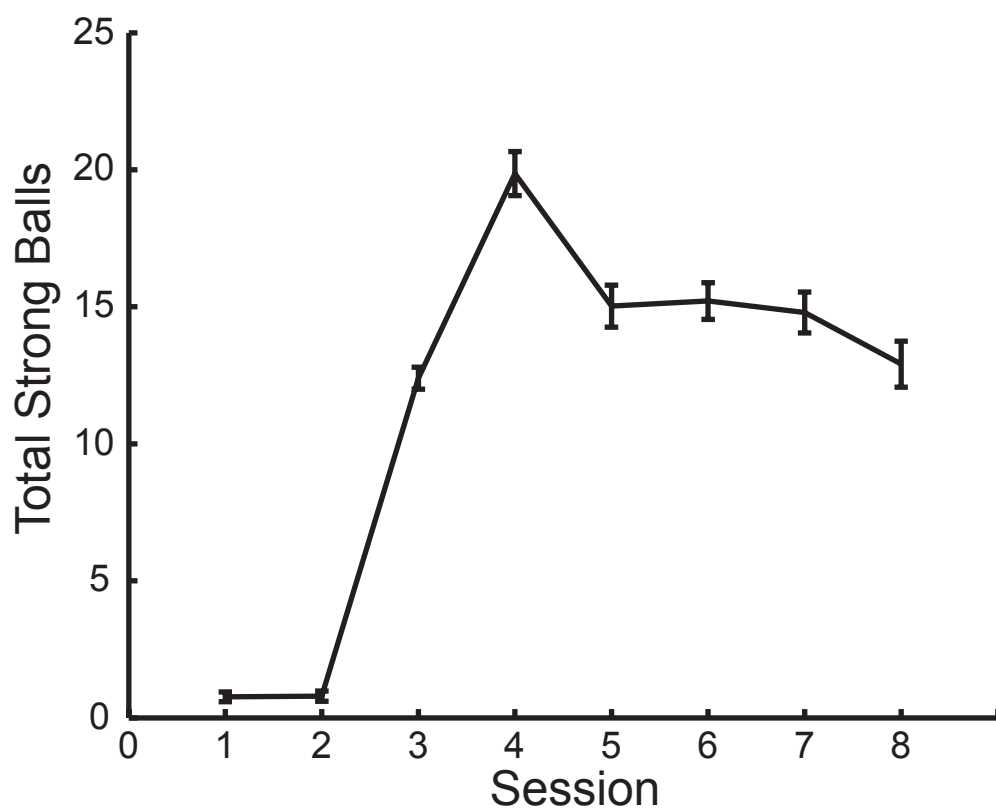

B

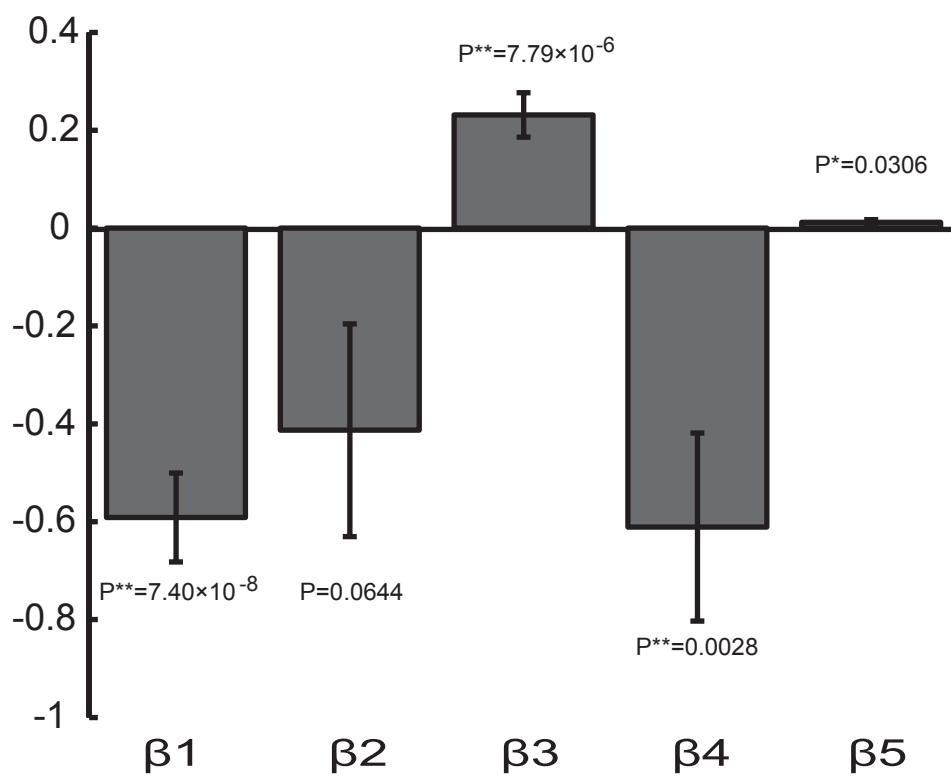

Supplement: scan-18-227-File008_nsy109 [file scan-18-227-file008_nsy109.pdf]
