# Supplementary material for: Behavioral and functional connectivity basis for peer-influenced bystander participation in bullying
Source: Soc Cogn Affect Neurosci. 2018 Nov 27;14(1):23–33. doi: 10.1093/scan/nsy109 (PMC6348439; doi:10.1093/scan/nsy109)
Supplement: Supplementary_Tables_nsy109 [file supplementary_tables_nsy109.docx]

**Supplementary Data**

**Supplementary Table 1.** Comparison of three optimal methods starting with different initial values. The average loglikelihood converged to the same value.

| Average negative log likelihood | |  |  |  |
| --- | --- | --- | --- | --- |
| initial value | [β1, β2, β3, β4, β5]= [ 0.1 0.1 0.1 0.1 0.1 ] | [β1, β2, β3, β4, β5]= [ 0.5 0.5 0.5 0.5 0.5 ] | [β1, β2, β3, β4, β5]= [ 2 2 2 2 2 ] | [β1, β2, β3, β4, β5]= [ 4 4 4 4 4 ] |
| interior-point | 63.75 | 63.75 | 63.75 | 63.75 |
| Sqp | 63.75 | 63.75 | 63.75 | 63.75 |
| active_set | 63.75 | 63.75 | 63.75 | 63.75 |

**Supplementary Table　2.** The 146 ROIs and MNI coordinates that were used for resting-state fMRI analysis. These ROIs constitute the gray balls depicted in Fig. 4A.

|  | ROI | MNI |  |  |
| --- | --- | --- | --- | --- |
| 1 | Dorsolateral_Prefrontal_Cortex_L | -45 | 26 | 24 |
| 2 | Dorsolateral_Prefrontal_Cortex_L | -27 | 36 | 31 |
| 3 | Dorsolateral_Prefrontal_Cortex_L | -10 | 47 | 36 |
| 4 | Anterior_Prefrontal_Cortex_L | -38 | 42 | 26 |
| 5 | Anterior_Prefrontal_Cortex_L | -32 | 50 | -1 |
| 6 | Anterior_Prefrontal_Cortex_L | -27 | 48 | 18 |
| 7 | Anterior_Prefrontal_Cortex_L | -16 | 60 | 3 |
| 8 | Anterior_Prefrontal_Cortex_L | -12 | 55 | -13 |
| 9 | Anterior_Prefrontal_Cortex_L | -10 | 57 | 20 |
| 10 | Anterior_Prefrontal_Cortex_L | -7 | 47 | 0 |
| 11 | Orbitofrontal_Area_L | -9 | 40 | -20 |
| 12 | Orbitofrontal_Area_L | -7 | 20 | -17 |
| 13 | Insular_Cortex_L | -39 | -11 | 13 |
| 14 | Insular_Cortex_L | -36 | 7 | -1 |
| 15 | Insular_Cortex_L | -35 | -12 | 0 |
| 16 | Inferior_Temporal_Gyrus_L | -49 | -11 | -26 |
| 17 | Inferior_Temporal_Gyrus_L | -57 | -43 | 7 |
| 18 | Inferior_Temporal_Gyrus_L | -56 | -12 | -10 |
| 19 | Inferior_Temporal_Gyrus_L | -56 | -29 | -13 |
| 20 | Inferior_Temporal_Gyrus_L | -54 | -27 | 3 |
| 21 | Superior_Temporal_Gyrus_L | -52 | 0 | 2 |
| 22 | Ventral_Posterior_Cingulate_Cortex_L | -8 | -56 | 21 |
| 23 | Ventral_Posterior_Cingulate_Cortex_L | -4 | -17 | 29 |
| 24 | Ventral_Posterior_Cingulate_Cortex_L | -4 | -33 | 32 |
| 25 | Ventral_Anterior_Cingulate_Cortex_L | -4 | 27 | 14 |
| 26 | Ventral_Anterior_Cingulate_Cortex_L | -4 | 4 | 24 |
| 27 | Ventral_Anterior_Cingulate_Cortex_L | -6 | -42 | 17 |
| 28 | Dorsal_Posterior_Cingulate_Cortex_L | -8 | -40 | 47 |
| 29 | Dorsal_Posterior_Cingulate_Cortex_L | -8 | -22 | 42 |
| 30 | Dorsal_Posterior_Cingulate_Cortex_L | -6 | -49 | 36 |
| 31 | Dorsal_Anterior_Cingulate_Cortex_L | -7 | 30 | 29 |
| 32 | Dorsal_Anterior_Cingulate_Cortex_L | -6 | 42 | 17 |
| 33 | Dorsal_Anterior_Cingulate_Cortex_L | -6 | 32 | -7 |
| 34 | Dorsal_Anterior_Cingulate_Cortex_L | -5 | 12 | 35 |
| 35 | Perirhinal_Cortex_L | -32 | -15 | -24 |
| 36 | Temporopolar_Area_L | -46 | 0 | -12 |
| 37 | Temporopolar_Area_L | -41 | 8 | -26 |
| 38 | Temporopolar_Area_L | -26 | 2 | -31 |
| 39 | Angular_Gyrus_L | -54 | -49 | 26 |
| 40 | Angular_Gyrus_L | -51 | -44 | 41 |
| 41 | Angular_Gyrus_L | -51 | -57 | 14 |
| 42 | Angular_Gyrus_L | -48 | -60 | 30 |
| 43 | Angular_Gyrus_L | -39 | -61 | 44 |
| 44 | Supramarginal_Gyrus_L | -57 | -28 | 33 |
| 45 | Supramarginal_Gyrus_L | -57 | -24 | 16 |
| 46 | Supramarginal_Gyrus_L | -55 | -38 | 23 |
| 47 | Supramarginal_Gyrus_L | -44 | -32 | 44 |
| 48 | Inferior_frontal_Gyrus_L | -52 | 15 | 14 |
| 49 | Inferior_frontal_Gyrus_L | -35 | 5 | 11 |
| 50 | Inferior_frontal_Gyrus_L | -51 | 29 | 6 |
| 51 | Inferior_frontal_Gyrus_L | -32 | 22 | 4 |
| 52 | Dorsolateral_Prefrontal_Cortex_L | -40 | 41 | 11 |
| 53 | Inferior_Frontal_Gyrus_L | -44 | 40 | -5 |
| 54 | Inferior_Frontal_Gyrus_L | -44 | 19 | -8 |
| 55 | Inferior_Frontal_Gyrus_L | -28 | 35 | -12 |
| 56 | Inferior_Frontal_Gyrus_L | -25 | 16 | -16 |
| 57 | Caudate_L | -14 | -15 | 20 |
| 58 | Caudate_L | -12 | 7 | 13 |
| 59 | Caudate_L | -11 | 18 | 2 |
| 60 | Putamen_L | -23 | 4 | 2 |
| 61 | Putamen_L | -10 | 4 | -6 |
| 62 | Thalamus_L | -18 | -35 | 15 |
| 63 | BA50_L | -10 | -26 | 3 |
| 64 | ParaHippocampal_L | -3 | -10 | 8 |
| 65 | Amygdala_AStr_L | -27 | -7 | -15 |
| 66 | Amygdala_CM_L | -21 | -6 | -14 |
| 67 | Amygdala_LB_L | -23 | -2 | -24 |
| 68 | Amygdala_SF_L | -15 | -5 | -19 |
| 69 | Hippocampus_L | -28 | -20 | -9 |
| 70 | Hippocampus_L | -27 | -38 | -1 |
| 71 | BA55.1_L | -4 | -24 | -9 |
| 72 | Dorsolateral_Prefrontal_Cortex_R | 6 | 45 | 21 |
| 73 | Dorsolateral_Prefrontal_Cortex_R | 14 | 48 | 36 |
| 74 | Dorsolateral_Prefrontal_Cortex_R | 24 | 48 | 25 |
| 75 | Dorsolateral_Prefrontal_Cortex_R | 37 | 37 | 26 |
| 76 | Dorsolateral_Prefrontal_Cortex_R | 50 | 23 | 27 |
| 77 | Anterior_Prefrontal_Cortex_R | 8 | 42 | 2 |
| 78 | Anterior_Prefrontal_Cortex_R | 13 | 60 | 0 |
| 79 | Anterior_Prefrontal_Cortex_R | 13 | 58 | 17 |
| 80 | Anterior_Prefrontal_Cortex_R | 29 | 52 | -5 |
| 81 | Anterior_Prefrontal_Cortex_R | 32 | 49 | 12 |
| 82 | Anterior_Prefrontal_Cortex_R | 43 | 43 | -2 |
| 83 | Orbitofrontal_Area_R | 5 | 30 | -6 |
| 84 | Orbitofrontal_Area_R | 6 | 41 | -17 |
| 85 | Orbitofrontal_Area_R | 8 | 24 | -19 |
| 86 | Orbitofrontal_Area_R | 11 | 53 | -16 |
| 87 | Insular_Cortex_R | 34 | 15 | -6 |
| 88 | Insular_Cortex_R | 39 | 4 | 4 |
| 89 | Insular_Cortex_R | 40 | -6 | 14 |
| 90 | Insular_Cortex_R | 41 | -11 | 0 |
| 91 | Inferior_Temporal_Gyrus_R | 36 | -16 | -25 |
| 92 | Inferior_Temporal_Gyrus_R | 46 | 0 | -29 |
| 93 | Inferior_Temporal_Gyrus_R | 46 | -53 | -33 |
| 94 | Middle_Temporal_Gyrus_R | 50 | -32 | 3 |
| 95 | Middle_Temporal_Gyrus_R | 55 | -14 | -20 |
| 96 | Middle_Temporal_Gyrus_R | 58 | -29 | -12 |
| 97 | Middle_Temporal_Gyrus_R | 62 | -40 | 0 |
| 98 | Superior_Temporal_Gyrus_R | 55 | -17 | -3 |
| 99 | Superior_Temporal_Gyrus_R | 56 | 0 | 2 |
| 100 | Superior_Temporal_Gyrus_R | 62 | -29 | 12 |
| 101 | Ventral_Posterior_Cingulate_Cortex_R | 6 | -42 | 19 |
| 102 | Ventral_Posterior_Cingulate_Cortex_R | 6 | -39 | 37 |
| 103 | Ventral_Posterior_Cingulate_Cortex_R | 7 | -18 | 30 |
| 104 | Ventral_Posterior_Cingulate_Cortex_R | 12 | -55 | 16 |
| 105 | Ventral_Anterior_Cingulate_Cortex_R | 7 | -6 | 43 |
| 106 | Dorsal_Posterior_Cingulate_Cortex_R | 6 | -54 | 31 |
| 107 | Dorsal_Posterior_Cingulate_Cortex_R | 9 | -28 | 45 |
| 108 | Dorsal_Posterior_Cingulate_Cortex_R | 17 | -63 | 27 |
| 109 | Dorsal_Anterior_Cingulate_Cortex_R | 6 | 11 | 30 |
| 110 | Dorsal_Anterior_Cingulate_Cortex_R | 7 | 30 | 17 |
| 111 | Perirhinal_Cortex_R | 11 | -43 | -17 |
| 112 | Perirhinal_Cortex_R | 15 | -43 | 3 |
| 113 | Temporopolar_Area_R | 29 | 0 | -32 |
| 114 | Temporopolar_Area_R | 37 | 12 | -24 |
| 115 | Temporopolar_Area_R | 50 | 1 | -13 |
| 116 | Angular_Gyrus_R | 33 | -67 | 38 |
| 117 | Angular_Gyrus_R | 48 | -59 | 34 |
| 118 | Angular_Gyrus_R | 48 | -57 | 17 |
| 119 | Angular_Gyrus_R | 58 | -47 | 24 |
| 120 | Supramarginal_Gyrus_R | 48 | -29 | 18 |
| 121 | Supramarginal_Gyrus_R | 49 | -31 | 49 |
| 122 | Supramarginal_Gyrus_R | 51 | -46 | 40 |
| 123 | Supramarginal_Gyrus_R | 57 | -20 | 31 |
| 124 | Supramarginal_Gyrus_R | 58 | -34 | 30 |
| 125 | Inferior_frontal_Gyrus_R | 36 | 17 | 29 |
| 126 | Inferior_frontal_Gyrus_R | 52 | 13 | 12 |
| 127 | Inferior_frontal_Gyrus_R | 35 | 19 | 8 |
| 128 | Dorsolateral_Prefrontal_Cortex_R | 46 | 37 | 13 |
| 129 | Inferior_Frontal_Gyrus_R | 18 | 12 | -15 |
| 130 | Inferior_Frontal_Gyrus_R | 26 | 33 | -13 |
| 131 | Inferior_Frontal_Gyrus_R | 43 | 24 | -11 |
| 132 | Inferior_Frontal_Gyrus_R | 52 | 25 | 3 |
| 133 | Caudate_R | 12 | 17 | 4 |
| 134 | Caudate_R | 14 | 0 | 17 |
| 135 | Putamen_R | 24 | 5 | 1 |
| 136 | Putamen_R | 29 | -8 | -1 |
| 137 | Thalamus_R | 5 | -13 | 7 |
| 138 | BA50_R | 8 | -25 | -7 |
| 139 | ParaHippocampal_R | 15 | -30 | 15 |
| 140 | Amygdala_AStr_R | 28 | -6 | -15 |
| 141 | Amygdala_CM_R | 23 | -5 | -14 |
| 142 | Amygdala_LB_R | 25 | -1 | -24 |
| 143 | Amygdala_SF_R | 18 | -4 | -18 |
| 144 | Hippocampus_R | 27 | -19 | -11 |
| 145 | Hippocampus_R | 30 | -36 | 0 |
| 146 | BA55.1_R | 8 | 1 | -4 |

**Supplementary Fig. 1** Behavior of players other than P2.

Session 1: P1, P3 and P4 only throw normal balls to other players with equal probability.

Session 2: P1 and P3 never throw a ball to P4. P1 and P3 only throws normal balls (blue arrows represent normal balls).

Session 3: P4 throws strong balls to others with equal probability. Red arrows represent strong balls. P1 and P3 throw only normal balls to P2 and P4.

Session 4: P1 starts to throw strong balls to P4 with a probability of 80%.

Session 5: In addition to P1, P3 starts to throw strong balls to P4 with a probability of 80%. This behavioral pattern continues in subsequent sessions.

**Supplementary Fig. 2** Four participants who suspected that other players might be computer programs were excluded from the data (n=39 in total). The main statistical results did not change by the exclusion.

**Supplementary Fig.3**

1. Total strong balls in each session. The graph shows the mean number of total strong balls from all players including participants. B) The session was incorporated as the fifth component of the utility function (equation (1’)). The bar graph shows the means of the estimated parameters.
